# Sanitary Pad Interventions for Girls' Education in Ghana: A Pilot Study

**DOI:** 10.1371/journal.pone.0048274

**Published:** 2012-10-31

**Authors:** Paul Montgomery, Caitlin R. Ryus, Catherine S. Dolan, Sue Dopson, Linda M. Scott

**Affiliations:** 1 Centre for Evidence Based Intervention, University of Oxford, Oxford, Oxfordshire, United Kingdom; 2 Said Business School, University of Oxford, Oxford, Oxfordshire, United Kingdom; Tehran University of Medical Sciences, Islamic Republic of Iran

## Abstract

**Background:**

Increased education of girls in developing contexts is associated with a number of important positive health, social, and economic outcomes for a community. The event of menarche tends to coincide with girls' transitions from primary to secondary education and may constitute a barrier for continued school attendance and performance. Following the MRC Framework for Complex Interventions, a pilot controlled study was conducted in Ghana to assess the role of sanitary pads in girls' education.

**Methods:**

A sample of 120 schoolgirls between the ages of 12 and 18 from four villages in Ghana participated in a non-randomized trial of sanitary pad provision with education. The trial had three levels of treatment: provision of pads with puberty education; puberty education alone; or control (no pads or education). The primary outcome was school attendance.

**Results:**

After 3 months, providing pads with education significantly improved attendance among participants, (lambda 0.824, F = 3.760, p<.001). After 5 months, puberty education alone improved attendance to a similar level (M = 91.26, *SD* = 7.82) as sites where pads were provided with puberty education (Rural M = 89.74, *SD* = 9.34; Periurban M = 90.54, *SD* = 17.37), all of which were higher than control (M = 84.48, *SD* = 12.39). The total improvement through pads with education intervention after 5 months was a 9% increase in attendance. After 3 months, providing pads with education significantly improved attendance among participants. The changes in attendance at the end of the trial, after 5 months, were found to be significant by site over time. With puberty education alone resulting in a similar attendance level.

**Conclusion:**

This pilot study demonstrated promising results of a low-cost, rapid-return intervention for girls' education in a developing context. Given the considerable development needs of poorer countries and the potential of young women there, these results suggest that a large-scale cluster randomized trial is warranted.

**Trial Registration:**

Pan African Clinical Trials Registry PACTR201202000361337

## Introduction

The positive links between female educational achievement and several measures of national wellbeing in underdeveloped countries have been well demonstrated [1]. Education predicts better economic productivity for women [1]. Furthermore, higher levels of education are also strongly correlated to key public health measures, including infant and maternal mortality, child nutrition, and early pregnancy [2], [3], [4]. Since the education acquired by girls is thought to have a significant impact on a community's future, their achievement is important on an absolute level. Of particular interest are the rapid reductions of fertility rates that seem to result from increased schooling for girls [2], [3], [4]; population growth has profound implications for economic wellbeing, social stability, and environmental protection [1], [2], [3], [4]. Thus, interventions that positively affect girls' enrolment and retention in schools are of keen interest in several policy arenas and academic disciplines, particularly where the betterment of poor nations is the focus.

Enrolment in primary education is considerably greater than that in secondary education and while positive outcomes resulting from primary level education are substantial, the magnitude of the outcomes is even greater when the impact of *secondary* schooling is considered [2], [4]. Addressing factors that compromise attendance and performance *throughout* the girl's school career may have far reaching implications for public health and development. A complex set of factors, involving gender norms and family practices, appear to be at work [5], [6], [7]. These include favoring investment in the male child, birth order, sexual activity, and arranged marriages [5], [6], [7], [8].

Culturally, the very event of menarche may signal to family and community that a girl is ready to be married and/or become sexually active, thus reducing her chances of staying in school [7], [9]. This tends to be coincident with the transition from primary to secondary education [7], [9]. Given the seeming salience of marriage and sexuality, and its timing with menarche, the possible role of menstruation as a barrier to, or negative influence on, schooling has recently become of interest [2], [3], [4]. Furthermore, practical concerns have emerged about the way basic amenities, such as poor toilet facilities, may impede menstruating schoolgirls [7], [9], [10], [11], [12], [13].

Observational studies have considered the socio-cultural implications of physical maturation and menstruation at an individual level, including taboos, myths, puberty rites, household responsibilities, and marital timing [5], [6], [8], [9]. These issues may all have a bearing on girls' school attendance. Some studies have directly observed this from self-report measures [9], [14], [15]. However, this research has not been used to develop interventions to mitigate any negative effects that the sociocultural values related to puberty may have on girls [9], [14], [15]. To date, NGO efforts have tended to focus on labor-intensive, long-term programs that aim to change views about the benefits of female education [10], [11]. High quality evidence of the efficacy of programs such as these is currently lacking. Since the public health, population, and economic benefits of female education are thought to occur within less than one generation [16], a short-term intervention with rapid impact of pad provision with education for girls might be useful.

**Figure 1:**
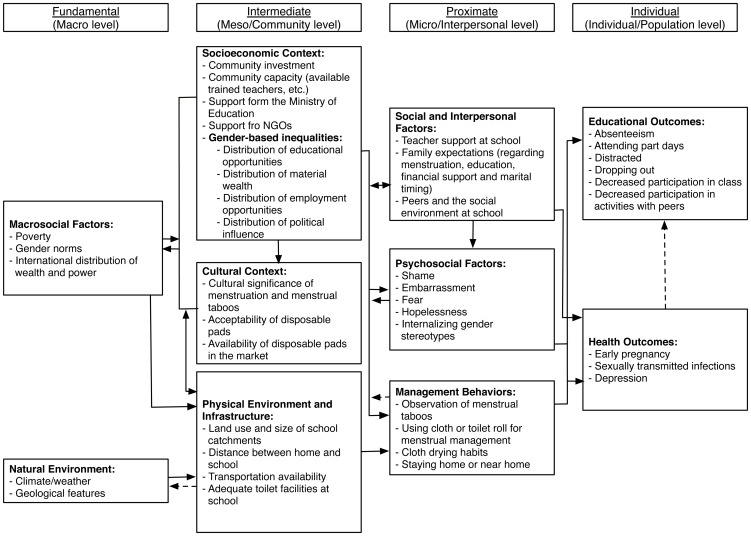
Ecological model of change as applied to health and educational outcomes of pads intervention.

This intervention might be expected to operate at multiple levels within an Ecological model of change [12], the full model is detailed in Figure 1.

There are more general background, “Macro” level obstacles to women's education, such as gender norms, poverty, the physical environment, and the lack of availability of sanitary pads in low and middle-income countries. The “Intermediate” level poses more specific obstacles to school attendance, including long distances between home and school and a lack of adequate and private toilet facilities there. The intervention at the “Proximate” level, aims to reduce shame, embarrassment, and stereotypes as well as responses such as using unsuitable materials for menstruation or staying at home. Outcomes are then measured at the Individual level, the primary one being attendance.

In this project, we designed and piloted such an intervention according to the MRC Framework for the Development and Evaluation of Complex Interventions to Improve Health [13]. To add greater understanding to the theory of change, qualitative investigations were also conducted which will be reported elsewhere [17].

### Aims

This study aimed to investigate the relationship between the availability of (1) sanitary pads-and-education and (2) education alone, on school attendance among girls aged 12–18 in Ghana.

### Hypotheses

1. The provision of Pads-with-education will improve attendance among menstruating schoolgirls.
2. The impact of the intervention will vary according to geographical location and associated levels of poverty. Rural areas will have better attendance rates than periurban areas upon provision of Pads-with-education.
3. Education about puberty and menstruation alone will not be sufficient to improve attendance at schools.

## Methods

The protocol for this trial and supporting CONSORT checklist are available as supporting information; see Checklist S1 and Protocol S1. In this we follow the reporting guidelines laid down by the TREND Statement [18]. The trial protocol was registered with the PAN African Trials Registry; the outcomes reported in this paper are the same as those that were measured from the start of the study. Ethical (IRB) approval was given by the Central University Research Ethics Committee at Oxford University and by the Ministry of Education in Ghana. Participant and parental informed consent was obtained for all participants. Research was conducted in two phases:

### Phase I: Feasibility and Development

This phase was a preliminary study to assess whether menstruation was indeed a barrier to education and to assess feasibility for the subsequent intervention phase. The researchers gathered information on local beliefs and practices concerning menstruation as well as cultural and infrastructural issues that could hinder school attendance for menstruating girls. Information was collected concerning how and from whom girls learned about menstruation; how girls managed their menses at home and in general; how well these methods worked at school; and the availability and quality of the toilet facilities in schools.

In late 2008/early 2009, with participation from local governmental and non-governmental organizations, research teams from Oxford went into the field. Semi-structured interviews with girls, parents, and teachers were conducted to understand conditions in the schools and to discuss the provision of sanitary care with education and health officials. All interviews were held in the relevant local language, with NGO staff providing interpretations into English. Fieldwork encompassed both Christian and Muslim populations in villages in both periurban and rural settings: Accra, as well as the Western, Central, Upper East, and Ashanti regions. Approximately 200 individuals drawn from each of the subject groups—girls, teachers, and parents—were interviewed through a combination of individual interviews, focus groups, and small community meetings. This was the maximum sample size possible with time and funding.

### Phase II: Piloting and Evaluation

#### Recruitment

Schools for the intervention were selected in collaboration with our NGO partner based on the criteria of having sufficient school population, a gender disparity in school enrollment, community acceptance, and enthusiastic support for the project. Gender disparity was used as a criterion to target schools where we were likely to find the problem for which the intervention was designed. Moreover, previous literature in this area and the intervention theory of change indicated that issues such as geography (rural, peri-urban) and religious affiliation (Christian, Muslim) would be important to investigate in a pilot study prior to a larger trial. Consequently, we consulted with our NGO partner “CARE” to ensure that sites incorporated a decent representation of these important factors given the limitations of this pilot study. Overall, we recruited four sites for the project. The periurban sites were selected to be comparable in terms of population density and economic development. The rural site was markedly less developed as the term usually suggests. After site recruitment, we used a coin flip to assign each site to a different intervention group.

The sample included 120 participants. At each site, teachers identified all the girls that were 12–18 at the school, and all girls meeting these criteria were referred by teachers to the study. Pre-menarcheal girls were excluded. No girls declined participation. Given the small size of each school community, all girls aged 12–18 at each school were included in the trial.

#### Intervention

The intervention had three levels of treatment each of 5 months duration:

1. provision of pads with puberty education (two sites)
2. puberty education alone
3. control site which received no pads or education (see Table 1).

**Table 1:** Percentage attendance across sites over time.

Treatment:	Location:	Pre-intervention attendance mean percent (SD)	Midpoint of intervention attendance mean percent (SD)	Post-intervention attendance mean percent (SD)
Pads-with-Education(n = 39)	Periurban	81.95 (29.06)	90.34 (10.52)	90.54 (17.37)
Pads-with-Education(n = 21)	Rural	77.09 (22.04)	92.43 (5.38)	89.74 (9.35)
Education only(n = 25)	Periurban	78.94 (24.81)	80.26 (19.01)	91.26 (7.82)
Control (n = 35)	Periurban	88.90 (12.40)	83.46 (12.50)	84.48 (12.4)

In each site there was a primary school and a junior secondary school. Girls in Pad-with-Education treatment sites were provided one pair of underwear in the event that they did not own any and twelve pads per month for the duration of the study. All participants received a daily calendar to record their menstrual cycles, as well as a pencil and sharpener. The educational component consisted of puberty education, including information regarding the development of secondary sex characteristics, the biological process of menstruation, and an explanation of how pregnancy occurs. Hygiene and menses management were discussed, and in treatment sites that received pads, girls were given instructions and demonstrations on how to use and dispose of the sanitary pads. Education was delivered in the local language at all three active intervention sites during school hours to groups of 15–25 girls by trained research assistants. These sessions were based on the curriculum used by the Ghana Education Service and were complemented with material from the Commonwealth Secretariat and Healthlink Worldwide ‘Gender and Relationships Handbook’ [15], which enabled consistent delivery.

Schools were offered gifts following participation valuing 250USD each. The monetary gifts were not used as incentives: these gifts were actually not promised in advance, but were presented after the study was over, as is culturally normal. This money was spent on goods that facilitated education. In one case toilet doors were purchased and installed. In another desks were repaired, and in a third, a water catchment system was installed. A school with no electricity had solar flashlights provided.

#### Context

Three sites were periurban villages in the Central region. Following consultation with our NGO partner, these sites were considered to be ethnically, economically, and culturally typical of periurban villages in the region. Results from Phase 1 had indicated that religious affiliation was not a key determinant in the factors under study and that other considerations were more important. In addition to the three main periurban sites, one Pads-with-Education treatment site was conducted at a remote rural location in the Ashanti region. This site was selected specifically because of its extreme remoteness; the location had no electricity, water, or paved roads, and no previous experience with sanitary pads.

#### Measures

Attendance was chosen as the primary outcome, as school performance data were hard to interpret across sites and grades. It is also likely that attendance acts as a suitable proxy measure for performance.

Teachers recorded their attendance daily as usual. Researchers collected attendance records dating from September 2008 to 2009. These dates captured 2 whole terms (1 term = 65 days) and one half term. Researchers compared official attendance data with actual student attendance at every site visit (planned and unplanned) and found negligible differences indicating strong reliability of the school attendance data.

Girls were provided with daily calendars and pencils with which to record their menstrual cycles and pad use. These data enabled researchers to assess the uptake of the intervention and implementation. Demographic data were collected and the girls were asked to report the time taken to walk to school and basic information concerning their menstrual history. An age-appropriate, culturally relevant poverty index was constructed to capture the multiple dimensions of poverty. Because accurate data on income and expenditures are difficult to obtain in a developing country context [14], an index of girls' material wellbeing was constructed using Asselin's indicators of household financial poverty that included ownership and/or access to common durable consumer goods (e.g. car, radio, TV, and phone); economic capital (land, livestock and income); water and sanitation (e.g. latrine at home); and the proximity of home to markets (i.e. distance to roads, shops, clinic, market). We also drew from the Oxford Poverty and Human Development Initiative [19] to capture noneconomic dimensions of girls' poverty, including wellbeing measures such as health; agency/empowerment; security (physical safety); shame; and subjective wellbeing. The latter measures have been found to be particularly accurate in research on adolescent and child health [20].

## Results

This was a three-armed study that tested a sanitary Pads-with-Education intervention versus education only versus no-intervention control for schoolgirls in Ghana to assess changes in school attendance. The mean age across the entire sample (n = 120) was 15.7 years (sd 1.59); this did not significantly differ between sites (H = 1.064 df = 3 p = 0.786). The average age of menarche was 14.08 (sd 1.91) and again did not significantly differ between sites (H = 4.73 df = 3 p = 0.192). All girls spoke either Twi or Fante and were all Christian, with the exception of two Muslim girls. The mean time distance to school was 0.6 hours (sd 0.55). There was a significant difference between sites (H = 32.137 df = 3 p<0.001) with the rural site having the longest time distance (1.3 h sd 0.8). Other sites were between 0.3 and 0.6 hours. The Poverty Index Scores also varied significantly across sites (H = 28.536 df = 3 p<0.001) with the rural site being the most impoverished (mean 2.67 sd 1.24) and the other sites between 3.68 and 5.2).

**Figure 2:**
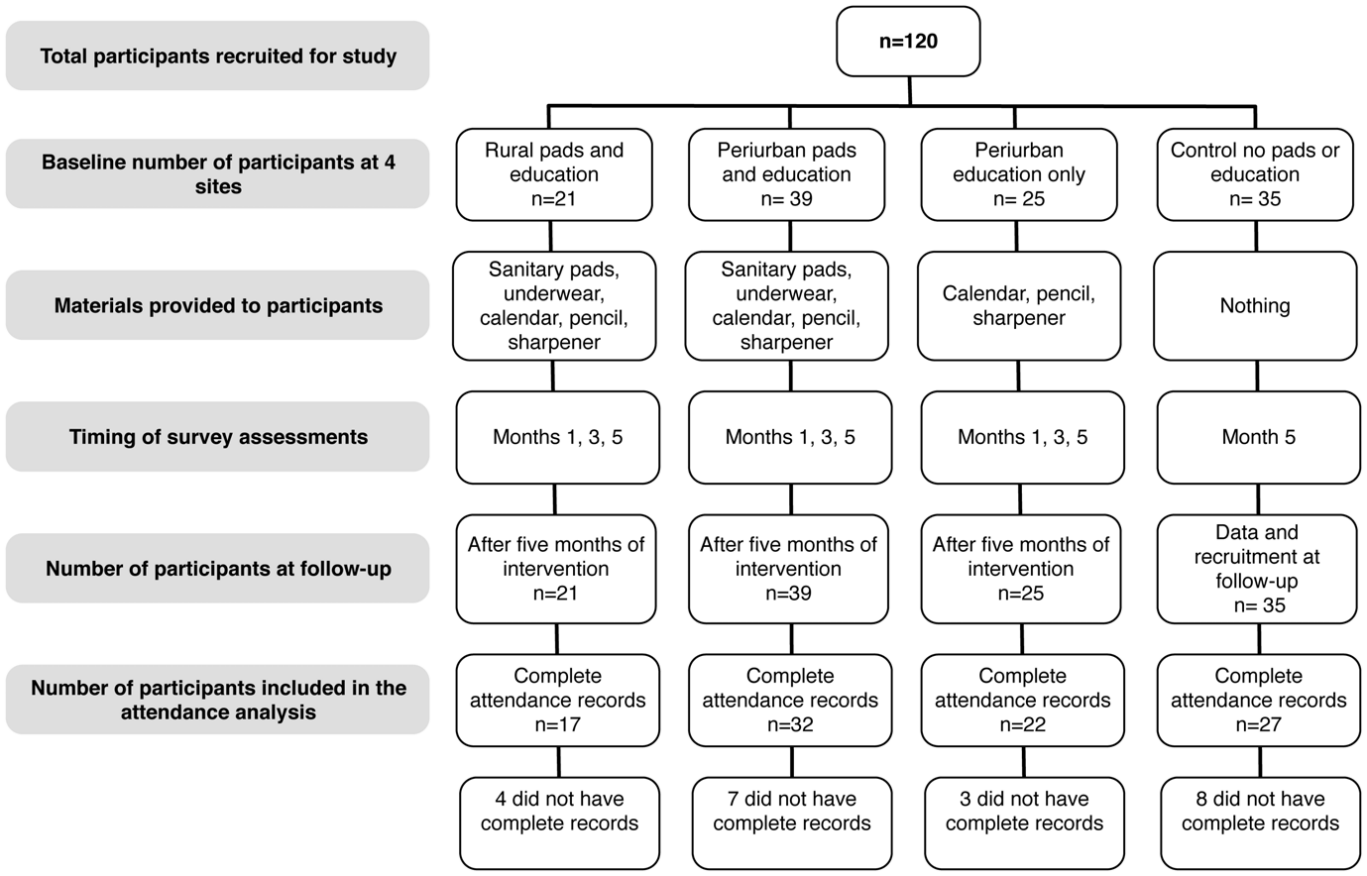
Participant flow chart.

**Figure 3:**
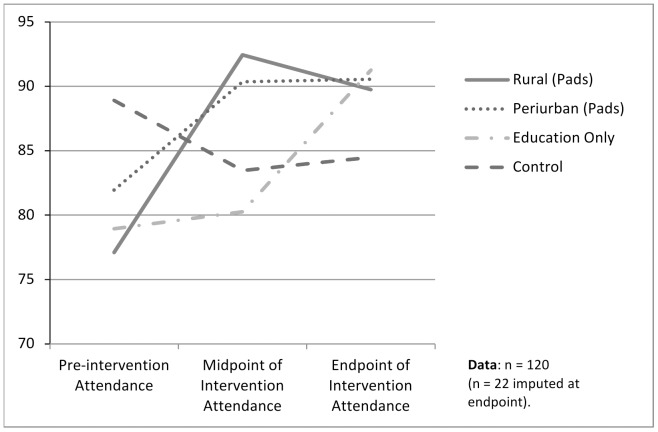
Percentage change in attendance by group over time.

The following analysis was conducted on an intent-to-treat basis. A participant flow chart across the four sites and three time points is provided in Figure 2. Missing values were mean-imputed with (Means: December = .83, April = .87, June = .89). There were 120 girls included in the attendance analysis (see figure 3). Of these 22 were missing complete attendance records, for these data were imputed.

In general, attendance rose in the Pads-with-Education groups by around 6 days per 65-day-term (or 9% of a girls' school year), this is illustrated in Figure 3. In the Education-Only group, initially, attendance did not change but at 5 months it rose to similarly high levels. There were few differences in attendance between active intervention sites and, in this respect, the rural site did not differ from its periurban counterpart. Detailed results can be found in Table 1.

At baseline (December 2008) the pupils in the four sites did not differ significantly, given the conventional 5% level with F(df) = 1.486 (3), p = 0.222.

After 3 months, providing pads with education significantly improved attendance among participants, (lambda 0.824, F = 3.760, p<.001). The changes in attendance at the end of the trial, after 5 months, were found to be significant by site over time. With puberty education alone resulting in a similar attendance level (M = 91.26, *SD* = 7.82) as sites where pads were provided with puberty education (Rural M = 89.74, *SD* = 9.34; Periurban M = 90.54, *SD* = 17.37), all of which were higher than control (M = 84.48, *SD* = 12.39).

Using PASW, a mixed between-within subjects analysis was conducted to assess the impact of the interventions at four sites (Pads-with-Education Rural, Pads-with-Education Periurban, Education-Only, and Control) on participants' absenteeism, across three time periods (pre-intervention, midpoint of intervention, post intervention). We used a repeated measures MANOVA, adjusting for the dependence of the observations (Huyuh-Feldt epsiolon = .811). With-in Subject Effects by side were significant with Huynh-Feldt's F(df) = 3.994 (4.865), p<0.002. The effect size (partial eta-squared) was 0.094, suggesting a moderate effect of the intervention.

An attempt was made to assess rural-urban differences on two sites which tested Pads-with-Education, one markedly rural, isolated, and lacking basic amenities, the other periurban. Attendance results at post-intervention were not significantly different between these sites (t = −0.232, df = 58, p = .845).

Findings from the qualitative phase of this work also suggested the potential for an intervention. Girls who had no access to sanitary pads reported missing 3–5 school days a month, which was corroborated by teachers and parents. These girls used “found” cloth instead of pads. They had limited access to soap and water to keep cloth clean and no private space for drying. Cloth was unreliable for the distance to school they had to travel and began to smell after a few days. They generally chose to stay home. Further information on menstruation practices will be included in a complementary paper on the qualitative investigations of this study [17].

## Discussion

This pilot evaluation is the first intervention study to assess the usefulness of sanitary pads with education for school attendance among menstruating students in Africa. Previous research in this area has been entirely observational, and thus the results of this controlled study are of interest to the research and international development community.

In accordance with the Ecological model, intermediate factors such as a longer distance from home to school, greater levels of poverty, and lack of water and toilets typical of rural areas are likely to make school attendance during menstruation more difficult. Thus, a greater effect at an individual level at our chosen rural site was predicted. However, girls at the rural site appeared to benefit to approximately the same degree as those at the periurban Pads-with-Education site. Based on these findings, it would seem that this intervention could be applied in a wider range of levels of urbanization. It may also be the case that these similar results suggest that the degree to which menstruation acts as a barrier to education is more closely related to cultural than geographical dynamics.

At the Education-only site, girls received the same educational material as at the Pads-with-Education sites except that they were not instructed on sanitary pad management. The effect of this minimal intervention was delayed but produced similar results to that of Pads-with-Education. That is, at a proximal level, it may be that the education component is the active ingredient across both interventions. Providing the girls with information and allowing discussion of this taboo subject may address factors at the interpersonal level of the ecological model such as management of cloth materials that enabled girls to better manage their periods, which perhaps resulted in increased school attendance. Alternatively, we may speculate that educating the girls in groups on this topic fostered improved peer and other relations making the school environment more supportive and in turn delivering the results presented here. Future research may consider study designs containing a “pads only” group in order to separate this effect from education.

Generalizability of these data may be limited in view of the sites chosen and population selected. However, many of the features and problems faced here are common to sub-Saharan Africa, and it is likely that this intervention would be worthy of testing elsewhere. In addition, given the limited number of sites located in different geographic settings, there is the possibility that results might be indicative of the particular site rather than the intervention assigned to that site. The sample may also have some selection bias in that participants included only those who were attending school despite menstruating, and thus had some basic internal and external resources in order to do so. Furthermore, we selected only students who had complete attendance records for three consecutive terms to provide comparative baseline data. It is possible that girls unable to fulfill this condition would be even more responsive to an intervention such as that presented here. Had they been included, we would expect that the effects of the intervention would have been greater.

Other limitations of this study include the small sample size and short follow-up time. This sample of 120 girls provides a reasonable template to begin to assess the issue of menstrual management and educational attainment. The short-follow-up time unfortunately does not allow us to determine the long-term effects of such an intervention. Acceptability of the intervention was not specifically tested in this per protocol analysis. However, qualitative data suggest that the girls found the education component of the intervention enjoyable and helpful, and there was no loss to follow-up for compliance reasons. A further consideration is that this intervention has cost implications and whether it is sustainable to scale is a long-term question for future studies. Nevertheless, for such pressing social, health and economic needs, these promising results require a well-conducted cluster randomized trial to assess the efficacy of pads and education for attendance and performance.

## Conclusions

This pilot evaluation is the first intervention study to assess the usefulness of sanitary pads with education for school attendance among menstruating students in Africa. The results suggest that provision of Pads-with-Education may be beneficial to improve attendance and the life chances of these young women. However, interestingly, Education-only also appears efficacious albeit with a delayed effect. We wish to highlight that this study requires replication as these results are exploratory only. Nonetheless, the overall patterns from this work indicate that further rigorous, effectiveness trial is warranted in accordance with the MRC Framework for Complex Interventions.

## Supporting Information

Protocol S1 **Trial protocol.**
(DOC)

Checklist S1 **CONSORT checklist.**
(DOC)
