# Supplementary material for: Sanitary Pad Interventions for Girls' Education in Ghana: A Pilot Study
Source: PLoS One. 2012 Oct 31;7(10):e48274. doi: 10.1371/journal.pone.0048274 (PMC3485220; doi:10.1371/journal.pone.0048274)
Supplement: Protocol S1 — Trial protocol. (DOC) [file pone.0048274.s001.doc]

**Full Proposal and Protocol**

**Girls’ Education and Sanitary Care in Developing Countries: An Exploratory Study**

*Linda Scott, Sue Dopson, Paul Montgomery, Catherine Dolan*

*University of Oxford, United Kingdom*

Introduction

The impact of female education on national development has been well demonstrated. Better schooling of girls not only leads to higher levels of productivity and expanded life chances in their adult years, but also has a dramatic impact on broader development goals, such as reduced fertility rates, lower infant mortality figures, and slowing HIV transmission (UNICEF 2007, World Bank, http://go.worldbank.org/1L4BH3TG20). Consequently, the international community is seeking mechanisms that will help keep girls in school in order to assist the development of poor nations, especially those in Africa. This study proposes a new tactic for retaining girls in school and would be executed in collaboration with Ghanaian partners, especially Plan Ghana and CARE Ghana. Letters of support from these organizations are appended.

There is a growing awareness that puberty, and menstruation in particular, can have an adverse effect on girls’ educational performance and constrain their long-term prospects to become productive members of society. In the past year, efforts to provide free sanitary towels for poor schoolgirls have received an increasing level of attention, as celebrity campaigns, cause-related television advertising in the US (see www.protectingfutures.com/home.jsp), and emergency airlifts into refugee camps drive home the development implications related to menstruation. Access to feminine care allows dignity and mobility to all menstruating women, but the salient emphasis on girls points to a growing view that the provision of sanitary protection improves school attendance among adolescent girls. Preliminary estimates suggest that lack of sanitary protection causes a significant number of girls to stay home from school a few days each month; they fall behind in their studies or become embarrassed and eventually drop out (UNICEF et. al. 2008).

Research Questions

1. Do appropriate facilities for managing menstruation [sanitary pads, private spaces, water, disposal mechanisms] improve female educational outcomes in developing countries? Which of these have the most impact?

2. How is reproductive health information, especially education about menstruation and puberty provided to young girls in developing nations and what group or individual barriers exist to their becoming informed?

3. Which institutional context [schools, families, churches, health care setting, community groups] provide the best mode of delivery for reproductive health information and sanitary products?

Project Background

Our team was approached in December 2007 by Procter & Gamble, the world’s largest manufacturer of feminine care products, with a request to collaborate on a large study in West Africa. P&G had previously partnered with UNICEF to research the impact of providing poor schoolgirls with free sanitary napkins and puberty education in Kenya and Malawi. This study was administered through UNICEF’s school program, and the plan was to “roll out” P&G’s sanitary care products and materials in poor nations everywhere, using UNICEF’s global schools presence. However, the research was poorly executed, and P&G sought a higher-quality, “independent” study that could potentially influence policy in poor nations around the world. At the same time, while we have been impressed by the commitment and vision of P&G, we agreed that we must pursue independent funding and control the development and execution of the research in order to ensure the objectivity of the study and the credibility of its finding in academic and policy circles.

Seedcorn Stage

Templeton College provided seedcorn funding for an initial scoping study of the issues, which was conducted in the spring of 2008. With this support, our team identified and met with key stakeholders (UNICEF, USAID, and Procter & Gamble in the US), conducted a review of relevant literature, assessed the results of the UNICEF research, invited an expert on African menstruation to Oxford for consultation, and carried out a preliminary investigation in Ghana to assess the feasibility and costs of an in-depth research project.

As the result of our learning from the scoping study, we are concerned that strategies that simply distribute manufactured sanitary towels through schools, even in conjunction with educational materials, may overlook key areas of potential intervention and lack cultural sensitivity. While we remain optimistic that providing sanitary products and, especially, information on puberty to poor schoolgirls will have a positive impact on their behaviour, potentially improving school attendance and sexual selectivity, we are unclear whether such interventions are generalizable across diverse contexts. We believe that local variations in attitudes and practices regarding menstruation as well as the range of conditions in schools, both of which would affect the outcome of a large-scale study, demand detailed qualitative investigations before supporting broad-based policy decisions. Further, we believe that alternative products, including locally available substitutes (some of which are more environmentally sound), should be investigated for comparative suitability in use.

We also feel that other institutions besides schools should be investigated as sites for the delivery of these materials. In many poor countries, the teachers are predominantly male, and, in several cases, sexual harassment of female students is a significant problem. Thus, the school setting may not be perceived as a safe environment in which to discuss such matters. Further, in countries where the dropout rate among females is very high, approaching girls through other venues may have a greater impact on reproductive outcomes, enabling the early identification of interventions that could prevent early dropout.

These concerns are sufficient to warrant an in-depth qualitative study that can provide the knowledge to inform design of a later, larger, randomized, trial. Our intention will be to use the learning derived from this Stage I to design a quantitative study, possibly with different delivery mechanisms, different information treatments, and even different products.

We have chosen Ghana as a field site for several reasons. First, African countries not only experience the lowest rates of female school attendance in the world, but the resulting negative outcomes (fertility rates, death in childbirth, HIV infection) significantly compromise their ability to improve their nations’ development prospects. Second, Ghana provides both Muslim and Christian communities for study—an important factor as religious attitudes toward women and menstruation are likely to produce significant differences. In particular, female genital cutting, still practiced at a low level in Ghana, will be an important concern, as these actions leave girls vulnerable to infection during menstruation. Third, the Government of Ghana has demonstrated a strong commitment to girls’ education, installing a Minister of Primary, Secondary and Girl-Child Education and creating a special unit within the Basic Education Division devoted to girls’ education. Finally, as mentioned, we already have access to some data about East and Southern Africa that will enable us to develop a comparative picture and draw preliminary generalizations across African countries.

To conclude, the scoping study supported by our seedcorn funds allowed us to formulate research questions firmly grounded in academic literature, policy discussions, and field observations in Ghana. This research has convinced us that feminine care is an important issue with implications for millions of girls and women worldwide—one that warrants subsequent rigorous and independent academic research.

Stage I: Qualitative Study

Based on the learning from the seedcorn stage we plan to conduct a qualitative investigation and feasibility assessment that will lay the foundation for the quantitative study planned for Stage II (described below). During Stage I, all members of the team propose to travel to Ghana at various times over a six-week period to:

♦ conduct interviews with girls, parents, nurses, teachers, community leaders;

♦ meet with government and NGO officials;

♦ review toilet facilities and sanitary provision at a variety of schools;

♦ visit community centres, churches, and clinics as possible alternative delivery sites for the intervention; and

♦ test feasibility of delivery methods and data collection.

We plan to conduct these activities at three key sites (Accra, Kumasi and Mankessim) that represent urban and rural as well as Muslim and Christian communities and show widely varying rates of female school enrolment. In each of the sites we propose collecting data from young girls through: 1) single-sex focus groups using participatory methods; 2) a semi-structured interview; and a 3) small cohort product trial. In addition, we plan to conduct semi-structured interviews with teachers, mothers, aunties, and grandmothers (2 each) in each of the three sites. Finally, at the end of Stage I we plan to initiate a small cohort study to assess the effectiveness of various sanitary products (described in the section: research tools). A final report of these activities and their outcome will be submitted to the Ethical Review Committee for review and approval prior to its dissemination and publication.

Research Collaboration

We have a team, both within Oxford University and on the ground in Ghana with Plan International and CARE International, which provides local expertise, as well as access to multiple sites (rural, urban, Muslim, Christian) and multiple institutional delivery systems (schools, community groups, churches, clinics).

Our own team, drawn from the Said Business School and the Centre for Evidence-Based Intervention at the Department of Social Policy and Social Work, who have experience in interviewing children safely. Our skill base includes experience with multinational companies and their inclusion of social welfare projects in the marketing/communications mix, international development and gender analyses of poverty, ethnography in Africa (including Ghana), organizational sociology in health care studies and knowledge translation issues, and evidence-based intervention, particularly with quantitative studies involving adolescent behaviour.

In Ghana, we plan to work closely with three local institutions: the Ministry of Education, the local CARE International team, and Plan International. CARE International has already initiated several girls’ empowerment projects in Ghana. Plan International has studied the impact of school latrine conditions on menstruating girls and is engaged in reproductive health training among young girls in several parts of the country.

Oxford University Staffing

**Linda Scott**, Professor of Marketing, Said Business School, specializes in markets, communication, and gender, with emphasis on consumer behaviour. She has extensive experience with multinational marketing companies that sell packaged goods to women.

**Catherine Dolan**, University Lecturer in Marketing, Culture, and Society, Said Business School, is an anthropologist specializing in the cultural and political economy of African development. She has conducted several research projects on gender, economic restructuring, and corporate responsibility in Africa, and has served as a consultant to the World Bank, DFID, USAID, and UNICEF.

**Sue Dopson**, Rhodes Trust Professor in Organisational Behavior, Said Business School, is an organizational sociologist specializing in healthcare studies and knowledge translation issues. She is a member of the Oxford Healthcare Management Institute.

**Paul Montgomery**, Reader in Evidence-Based Interventions, is trained in child/adolescent psychiatry and specializes in the analysis of complex interventions. He has past clinical experience as a child and family therapist.

Bibliography:

Buckley, Thomas and Alma Gottlieb (1988), *Blood Magic: The Anthropology of Menstruation*, Berkeley: University of California.

Burrows, A. and S. Johnson (2005), “Girls’ Experiences of Menarche and Menstruation”, *Journal of Reproductive and Infant Psychology*, 23 (3), pp. 235-249.

Delaney, Janice, Mary Jane Lupton and Emily Toth (1988), *The Curse: A Cultural History of Menstruation*, Urbana: University of Illinois.

Douglas, Mary (1966), *Purity and Danger: An Analysis of Concepts of Pollution and*

*Taboo,* London: Routledge & Kegan Paul.

El-Gilany, Abdel-Hady, Karima Badawi and Sanaa El-Fedawy (2005), “Menstrual Hygiene among Adolescent Schoolgirls in Mansoura, Egypt,” *Reproductive Health Matters,* 13 (26): 147-152.

Fakeye, O. and A. Adegoke (1994), “The Characteristics of the Menstrual Cycle in Nigerian School Girls and the Implications for School Health Programmes,” *African Journal of Medicine and Medical Sciences*, 23 (1): 13-17.

Ghana, Ministry of Education, “Girl’s Education in Ghana: Trends, Initiatives, and the Way Forward,” Presentation.

Hausmann, Ricardo, Laura D. Tyson and Saadia Zahidi (2007), *The Global Gender Gap Report 2007*, Geneva: World Economic Forum.

Houppert, Karen (2000), *The Curse: Confronting the Last Unmentionable Taboo*, New York: Farrar, Strauss, and Giroux.

Kirk, Jackie and Marni Sommer (2006), “Menstruation and Body Awareness: Linking Girls’ Health with Girls’ Education,” http://www.schools.watsan.net/content/download/323/2726/

file/Kirk-2006-Menstruation-KIT%20paper.pdf.

Knight, Christopher (1995), *Blood Relations: Menstruation and the Origins of Culture,* New Haven, Conn.: Yale University Press.

Madhaven, S. and A. Diarra (2001), “The Blood that Links: Menstrual Regulation Among the Bamana of Mali,” in Etienne van de Walle and Elisha Renne (eds.), *Regulating Menstruation: Beliefs, Practices, Interpretations*. Chicago: University of Chicago Press, 172-186.

Martin, E. (1998), “Medical Metaphors of Women’s Bodies: Menstruation and Menopause,” in P. J. Brown (ed.), *Understanding and Applying Medical Anthropology*. Mountain View, Calif.: Mayfield, pp. 345-356.

Matasha E, Ntembelea T, Mayaud P, Saidi W, Todd J, Mujaya B, and L. Tendo-Wambua (1998), “Sexual and Reproductive Health among Primary and Secondary School Pupils in Mwanza, Tanzania: Need for Intervention,” *AIDS Care*, 10 (5): 571-82.

Mbizvo, MT, J. Kasule, V. Gupta, S. Rusakaniko, J. Gumbo, SN Kinoti, W. Mpanju-Shumbusho, Sebina-Zziwa, R. Mwateba, and J. Padayachy (1995), “Reproductive Biology Knowledge, and Behaviour of Teenagers in East, Central and Southern Africa: The Zimbabwe Case Study,” *Central Africa Journal of Medicine*, 41 (11): 346-54.

Mooljman, Annemarieke (2007), *Middle Childhood: Children 6/7-12 Years: Guidelines and Tools on Hygiene, Sanitation and Water in Schools*, Plan International (West Africa Region).

Nabar, Qumrun and Rokeya Ahmed (2006), “Addressing Special Needs of Girls: Challenges in School,” Presented in SACOSAN II, 2006 at Islamabad, Pakistan.

Ngom, P. (2000), “Stomach Washing: Menstrual Inducement among the Kassena-Nankana of Northern Ghana,” *African Population Studies* 15 (1): 109-16.

Plan International, “Girl-Friendly Toilets for Schoolgirls,” http://www.schools.watsan.net/content/download/329/2772/file/nine

Ross, David A., John Changalucha, Angela I. N. Obasi, Jim Todd, Mary L. Plummer, Bernadette Cleophas-Mazige, Alessandra Anemona, Dean Everett, Helen A. Weiss, David C. Mabey, Heiner Grosskurth, and Ricahrd J. Hayes (2007), “Biological and Behavioural Impact of An Adolescent Sexual Health Intervention in Tanzania: A Community Randomized Trial,” *AIDS*, 21: 1943-1955.

Sommer, Marni (2008), “Where the Educational System and Women's Bodies Collide: The Social and Health Impact of Girls' Experiences of Menstruation and Schooling in Tanzania,” Unpublished dissertation, Columbia University Mailman School of Public Health.

UNICEF, 2007, The State of the World’s Children 2007 http://www.unicef.org/sowc07/docs/sowc07.pdf

UNICEF, Procter & Gamble, FAWE Kenya, and the Ministry of Education of Kenya (2008), “The Impact of Feminine Hygiene on Girls’ Participation in Education: A Study of Primary Schools in Garissa and Dagoretti Districts,” Unpublished study.

van de Walle, Etienne and Elisha P. Renne (2001), *Regulating Menstruation: Beliefs, Practices, Interpretations*, Chicago: University of Chicago Press.

**Summary Proposal and Protocol**

Girls’ Education and Sanitary Care in Developing Countries: An Exploratory Study

Oxford University

The impact of female education on national development has been well demonstrated. Better schooling of girls not only leads to higher levels of productivity and expanded life chances in their adult years, but also has a dramatic impact on broader development goals.

There is a growing awareness that puberty, and menstruation in particular, can have an adverse effect on girls’ educational performance and constrain their long-term prospects to become productive members of society. Preliminary estimates suggest that lack of sanitary protection causes a significant number of girls to stay home from school a few days each month; they fall behind in their studies or become embarrassed and eventually drop out.

Research Questions

1. Do appropriate facilities for managing menstruation [sanitary pads, private spaces, water, disposal mechanisms] improve female educational outcomes in developing countries? Which of these have the most impact?

2. How is reproductive health information, especially education about menstruation and puberty provided to young girls in developing nations and what group or individual barriers exist to their becoming informed?

3. Which institutional context [schools, families, churches, health care setting, community groups] provide the best mode of delivery for reproductive health information and sanitary products?

We propose to:

♦ conduct interviews with girls, parents, nurses, teachers, community leaders;

♦ meet with government and NGO officials;

♦ review toilet facilities and sanitary provision at a variety of schools;

♦ visit community centres, churches, and clinics as possible alternative delivery sites for the intervention; and

♦ test feasibility of delivery methods and data collection.

We plan to conduct these activities at three key sites (Accra, Kumasi and Mankessim) that represent urban and rural as well as Muslim and Christian communities and show widely varying rates of female school enrolment. In each of the sites we will collect data from young girls through: 1) focus groups using participatory methods; 2) a semi-structured interview; and a 3) small cohort product trial. In addition, we plan to conduct semi-structured interviews with teachers, mothers, aunties, and grandmothers (2 each) in each of the three sites.

**Informed Consent Form**

**Girls’ Education and Sanitary Care in Developing Countries: An Exploratory Study**

**Investigators Name:** Linda Scott, Catherine Dolan, Sue Dopson, and Paul Montgomery

**Affiliation:** Said Business School and Center for Evidence-Based Intervention, Oxford University

We are inviting you to take part in a six week research study that we are conducting on menstruation and sanitary care among young women in Africa. If you are willing to participate in the study, we would like to obtain your consent.

We are asking you to participate in this study because we would like to understand how girls experience menstruation in Ghana and whether they have adequate toilets and sanitary care at home and at school. If you decide to take part in our study, we will ask you to participate in an interview or in an informal group discussion with other members of your school and/or community. We will either come to your home or meet you in a place that is convenient for you. These discussions will also be approximately one hour in length and will be scheduled at a time that suits you.

There are no risks to you or your family from your participation in this study. Your participation in the study will also remain completely confidential. We will assign a code to your name that only we know and store this information in a computer that is password protected. That means no one except us will know that the answers you give are from you. No reports or publications will use information that can identify you in any way. In addition, we would like your permission to tape record the interview so that they can be transcribed at a later date. They will be destroyed following the transcriptions. Once we have completed the study we will work with Plan International and/or CARE International to ensure that you are informed about the results of this research.

It is important that you realize that you do not have to participate in the study if you do not want to. Your participation is completely voluntary. You are also free to decline any or all questions, and withdraw from the study at any time. While some of these questions may seem embarrassing to you, we will do everything we can to ensure that you feel comfortable.

If you have any questions about the research, we would be happy to discuss these with you now. And if you have any questions or problems related to the research, you can contact us or our relevant partners (Plan International and CARE International) in Ghana at the numbers provided below.

**Do you agree to participate in this study and understand that you are free to withdraw from the study at any time? (Interviewer circles respondent answer)**

**(1) Yes**

**(2) No**

__________________________________________________ _________

Signature of person who explained the study to the participant Date

**Contact Details:**

1) Linda Scott, Said Business School, University of Oxford, Oxford UK OX1 1HP

Telephone in Ghana: 02730448822. Email address: linda.scott@sbs.ox.ac.uk.

2) Bright Wireko-Brobby, CARE Gulf of Guinea, Kumasi Sub Office
P. O. Box AS 18, Asawase, Kumasi
Telephone: 051-61886/7, Cell: 024-4837 822

3) Emelia Allen, Plan Ghana (Telephone: 024-3132829) or Gloria Obeng-Amoako, Plan Ghana (0243178009)

**Questionnaire and Research Tools**

In the qualitative study we aim to conduct research with various stakeholders including young girls, as well as their teachers and female relatives.

**A. Data Collection: Young Girls**

We propose to collect data from young girls through three primary methods: 1) focus groups employing participatory tools; 2) semi-structured interviews; and 3) a cohort study.

**1. Focus Groups using Participatory Methods**

In each of the three study locations we plan to conduct four single-sexed focus groups (approximately 6 - 8 girls in each group) that use participatory methods to elicit involvement and open discussion. Participatory methods have been recommended as one of the most appropriate ways to facilitate discussions between adults and children by the Girls’ Education Unit of the Ghana Education Service. They are specifically designed to be child-centred and are important in recognizing the agency of children/adolescents as individuals in their own right. By eliciting the ‘authentic’ perspectives of children/adolescents, such methods are not only often more culturally sensitive but can also help to ensure that children/adolescents actively shape the research process and its subsequent outcomes.

We propose using visual research tools to elicit the perspectives of adolescent girls and generate insights on how gender differences implicate reproductive health and their experience of menstruation in particular. This will involve showing the girls a series of pictures, including the following, as catalysts for discussion:

♣ A girl sitting at home, watching boys go off to school.

♣ A girl sitting in school looking anxious.

♣ A girl walking to school.

♣ A girl sitting in school looking confident and/or a girl standing to answer questions in school.

♣ A girl walking to the toilet at school.

♣ A girl-friendly toilet.

**2. Semi-Structured Interviews (SSIs)**

We also plan to conduct a semi-structured interview with 10 individual girls across the three study locations (30 interviews in total) in order to obtain baseline knowledge of how menstruation is experienced. The following is a preliminary draft of the questionnaire.

**Semi-Structured Questionnaire for Adolescent Girls**

*Girls’ Education and Sanitary Care in Developing Countries: An Exploratory Study*

**I. Socio-Economic and Demographic Information**

1. ID number of respondent:____________

2. Location: _____________

3. Age: __________

4. Religion: _______________

5. Are you currently in school? Yes (specify current grade/class level) ____ No____

6. If yes, how long does it take you to walk to school each day? _______

7. If no, what is the highest level of education that you have completed________?

8. How many people live in your household?

| **Household members** | **Total Number** |
| --- | --- |
| Self |  |
| Brother |  |
| Sister |  |
| Mother |  |
| Father |  |
| Grandparents |  |
| Aunts |  |
| Uncles |  |
| Other (Specify) |  |
| **Total household members** |  |

9. Does your household own or have the following:

|  | **Yes** | **No** |
| --- | --- | --- |
| Electricity | 1 | 2 |
| Running water inside the house | 1 | 2 |
| Flush toilet inside the house | 1 | 2 |
| Land | 1 | 2 |
| Livestock | 1  2 |  |
| Car | 1  2 |  |
| Television | 1 | 2 |

10. If you do not have running water in your house, where do you obtain it? (note distance from house)

_____________________________________________________________________

11. If you do not have a flush toilet inside your house, what sort of toilet/latrine do you use?

_____________________________________________________________________

12. If you dispose of garbage at home, what method do you use?

a. Burning

b. Composting

c. Community Garbage Collection Point

d. Other __________________

**II. Feminine Hygiene**

13. Have you had your period yet?

14. At what age did you begin to menstruate?

15. How many days does your period typically last each month? ________

16. Who gave you your first information about menstruation? (Tick all that apply)

| Teacher at school |  |
| --- | --- |
| Doctor/gynaecologist |  |
| Nurse |  |
| School clubs |  |
| Friend |  |
| Mother |  |
| Sister |  |
| Aunt |  |
| Grandmother |  |
| Articles in newspapers/magazines |  |
| Other (specify) |  |

17. Before you started to menstruate, what did you know about it?

________________________________________________________________________

________________________________________________________________________

18. Can you describe what happened during your first period?

________________________________________________________________________

________________________________________________________________________

19. How would you rate your knowledge about puberty? (circle the correct answer)

| Poor | Fair | Good | Very Good | Excellent |
| --- | --- | --- | --- | --- |

20. How would you rate your knowledge about menstruation? (circle the correct answer)

| Poor | Fair | Good | Very Good | Excellent |
| --- | --- | --- | --- | --- |

21. Are there people that you feel free to discuss your period with? If so, who and why?

________________________________________________________________________

22. Are there people that you do not want to tell that you are menstruating? If so, who and why?

________________________________________________________________________

23. Does menstruation ever cause you to…(please tick ALL that apply or add your own)

| Miss school / education |  |
| --- | --- |
| Miss work |  |
| Be unable to carry out daily activities (specify) |  |
| Be unable to participate in sports |  |
| Feel ashamed |  |
| Feel isolated from others |  |
| Feel frustrated |  |
| Feel upset |  |
| Other |  |

24. Which of the following issues (if any) did you experience during your last period? (Tick all that apply)

| Skin irritation / rashes to the body |  |
| --- | --- |
| Felt insecure |  |
| Panty soiling |  |
| Outside garment soiling |  |
| Sticky/sweaty feelings |  |
| Wet feeling |  |
| Unpleasant odor |  |
| Headache |  |
| Cramps |  |
| Boating/tenderness/swelling |  |
| Back pains |  |
| Irritability/moodiness |  |
| Depression |  |
| Other (specify) |  |

25. How much do agree/disagree with the following statements? (Check the appropriate column)

| Strongly Agree | Somewhat Agree | Neither Agree nor Disagree | Somewhat Disagree | Strongly Disagree |  |
| --- | --- | --- | --- | --- | --- |
| I feel insecure during my period |  |  |  |  |  |
| Period days are like any other day (I do not feel any difference) |  |  |  |  |  |
| I prefer staying at home during my period |  |  |  |  |  |
| During my period I feel less self-confident than during other days |  |  |  |  |  |
| During my period I avoid physical activity |  |  |  |  |  |
| I am afraid of my next period |  |  |  |  |  |
| I wish I would know more about my period |  |  |  |  |  |
| Boys tease me about my period |  |  |  |  |  |

26. Have you been circumcised? Yes ____No_____

27. If yes, has this affected your periods in any way? (e.g. longer or more painful periods)

_____________________________________________________________________

28. What do you and other women in your household use to manage your mense flow? (Please circle whether you use it often, sometimes, or never)

a. Reusable Manufactured Sanitary Pads Often Sometimes Never

b. Disposable Sanitary Pads Often Sometimes Never

c. Tampons Often Sometimes Never

d. Sponges Often Sometimes Never

e. Rags/Old Clothes Often Sometimes Never

f. Cotton Wool Often Sometimes Never

g. Other (e.g natural product / local material)

Please state________________ Often Sometimes Never

29. If you know the name of the product you use, please circle below:

a. Stayfree

b. Always

c. Tampax

d. Kotex

e. Forever Easy

f. All One*

g. Smart Lady*

h. All Easy*

i. Other (Please state) _______________________

30. If you use a sanitary pad, how many do you use per day (average)? _____

31. How important are the following for you in a sanitary pad? Please circle you response.

*(Not Important* 1 2 3 4 5 *Very Important)*

a. Washable 1 2 3 4 5

b. Disposable 1 2 3 4 5

c. Environmentally Friendly 1 2 3 4 5

d. Made from local product 1 2 3 4 5

e. Leak Free 1 2 3 4 5

f. Comfortable 1 2 3 4 5

g. Discrete 1 2 3 4 5

h. Low Cost 1 2 3 4 5

i. Sold at convenient locations 1 2 3 4 5

j. Allow freedom of movement 1 2 3 4 5

32. Do you think that your current method / product used to manage your period is effective?

Yes ____

No____

No opinion ____

33. Please explain your response.

______________________________________________________________________

______________________________________________________________________

34. How happy are you with what you currently use? (circle)

*Not happy at all* 1 2 3 4 5 *Very Happy*

35. Please explain your response.

36. Who is responsible for buying / providing you with sanitary towels or any other product you use during your period? (tick)

| Yourself |  |
| --- | --- |
| Family member (specify) |  |
| Friend |  |
| Other (specify) |  |

37. Where does this person usually buy or obtain the product? (tick those that apply)

| Local pharmacy |  |
| --- | --- |
| Local shop |  |
| Market |  |
| Other women |  |
| NGO |  |
| Hospital or Clinic |  |
| School |  |
| Other (specify) |  |

38. If you purchase these products yourself do you feel shy about buying or asking for them? (please state why or why not)

______________________________________________________________________

39. Do you know how much the product you use costs? Yes ____ No ____

40. If yes, please specify _______ per _______

*For example: 5000 GHc per pad*

*50,000 GHc per menstrual cycle*

41. Have you ever had difficulty paying this price? (circle)

Yes No No Opinion

42. If yes, are there things that you do not buy so that you can purchase a sanitary product? Yes (specify)__________ No______

43. (If they currently use a global brand): Would you be interested in trying a locally-produced sanitary pad? Yes ____ No ____ Maybe _____

Why / why not? ___________________________________

44. Do you wear underpants?

Yes_____ No______

**School-related Information**

45. Can you access water at your school? Yes____ No_____

46. If yes, what is the source? (e.g. running water tap, borehole, stream etc)____

47. Is there a toilet/latrine at school? Yes ____ No ____

48. If yes, do you share this with boys? Yes____ No ____

49. How many days of school did you miss in the past three (3) months because of problems related to your period?

_____________________________________________________________________

50. Would you prefer to go to school or stay home when you are menstruating? Explain in either case.

____________________________________________________________________

51. Do you change your sanitary pad/menstrual protection at school? Yes ____ No _____

52. If yes:

a) Where do you change? ______________________________

b) Do you have privacy? _______________________________

c) Is there water available for washing your hands? __________

d) Where do you dispose of your used pad? _________________

53. What do you do if you soil your clothes during school?

________________________________________________________________________

54. How would you help a friend who had soiled her clothes (from menstruation) during school?

_____________________________________________________________________

55. Do any of the teachers at school assist you when you are menstruating? Yes ___ No____

56. If yes, what sort of assistance do they provide (e.g. allowing more bathroom breaks etc).

____________________________________________________________________

57. If you were the head teacher of your school, which, from the following list, would you give priority to first? (Rank)

| Stocking sanitary towels in the school |  |
| --- | --- |
| Allow girls to answer questions while seated to avoid embarrassment in case of soiling one’s dress |  |
| Ensure plenty of water to allow girls to clean themselves |  |
| Ensure that the girls’ toilets are far from the boys to allow for privacy |  |
| Ensure that there are disposal bins and incinerators for disposing used sanitary towels |  |
| Ensure that there are remedial classes for the girls who miss class because of menstruation |  |

Additional Comments:

**3. Cohort Study**

Upon the completion of the Stage I semi-structured interviews and participatory research, we plan to implement a small cohort study that tests the effectiveness of various options for sanitary care. This will involve dividing 100 girls into 5 groups. Each group will be given one brand of sanitary protection (selected from the 5 leading products identified in the semi-structured interviews) to test for one menstrual cycle. Following the test, each girl will be asked a short set of questions related to the effectiveness, absorption, durability and comfort of the pad.

**B. Data Collection: Teachers**

In each of the three study locations we plan to interview two teachers to gain an understanding of: 1) the challenges that menstruation poses for female educational attainment; 2) the limitations (if any) of current sanitary facilities at school; 3) the current role staff and teachers play in facilitating puberty education; and 4) recommendations for improving menstrual management and puberty education in the school environment.

**C. Data Collection: Female Relatives**

In each of the three locations we plan to interview the mothers, aunties and grandmothers of the girls in our SSI sample (2 of each category in each location). These interviews will be conducted to gain an understanding of the socio-cultural position of menstruation in the three locations and in particular, how puberty and menstruation are understood, practiced and managed by women across life stages. The interviews will include an in-depth discussion of how menstruation is (and has been) historically perceived (celebrated or stigmatized); what methods are used to manage menstrual blood; and how knowledge related to reproduction, puberty and menstruation are (or are not) transmitted inter-generationally between women.

**Linda M. Scott**

Curriculum Vitae

**Education**

University of Texas at Austin; Ph. D., Communications; 1991.

Southern Methodist University; Master of Business Administration; 1978.

University of Texas at Austin; Master of Arts, English (American Literature and Culture); 1976.

University of Texas at Austin; Bachelor of Arts, English (and History); 1974.

**Academic Positions Held**

Professor of Marketing, Oxford University, Said School of Business, August 2008 to present.

Reader in Marketing, Oxford University, Said School of Business, June 2006 to July 2008.

Associate Professor of Advertising, Art & Design, and Women’s Studies, Research Associate Professor, Institute for Communications Research, University of Illinois.

Assistant Professor, School of Mass Communications, University of Colorado, 1991-1992.

**Selected Publications**

Linda M. Scott (2007), “Critical Marketing: An Armchair Report,” *Critical Marketing*, Pauline McLaran and Avi Shankar, eds. (London: Elsevier).

Linda M. Scott, Jason P. Chambers, and Katherine Sredl (2007), “The Monticello Correction: Consumption in History,” *Handbook of Qualitative Research Methods in Marketing*, Russell Belk, ed. (UK: Cheltham, Gloucestershire, in press).

Linda M. Scott (2005), *Fresh Lipstick: Redressing Fashion and Feminism* (New York: Palgrave MacMillan).

Linda M. Scott (2003), “Introduction,” and Interviews with Gloria Steinem and Mary Lou Quinlan, *Advertising & Society Review*, special issue on women in advertising, ed. Linda Scott (December).

Linda M. Scott (2000), “Market Feminism: The Case for A Paradigm Shift,” *Marketing and Feminism,* edited by Miriam Catterall, Lorna Stevens, and Pauline MacLaren, London: Routlege (lead chapter).

Scott, Linda M. (1993), "Fresh Lipstick: Rethinking the Images of Women in Advertising," *Media Studies Journal*, Winter/Spring, 144-155.

Tharp, Marye and Linda M. Scott (1990), "The Role of Marketing Processes in Making Cultural Meanings," *Journal of Macromarketing*, Fall, 47-60.

**Recent Invited Presentations**

“Avon in Africa,” Notre Dame University, November 2008.

“Avon in Africa,” University of Exeter, March 2008.

“Globalization and the Goddess: Women’s Empowerment through Markets,” Omnicom, New York City, November 2007

“Subversive Consumption: 19^th^ Century Irish Immigrants to America,” Consumer Identity Group Symposium on “Finding an Irish Voice,” Dublin City University, October 2007.

“Avon in Africa,” EdHec, Lille, France, October 2007.

“The Fashion Forward Feminist,” Rice University, Houston, Texas, October 4, 2006.

“Fresh Lipstick” and “The New Femininity,” Virginia Tech, Blacksburg, VA, April 2006.

“Fresh Lipstick,” University of Colorado at Boulder, March, 2006.

“Materialism and Gender,” Aspen Chapel, Aspen, Colorado, March 2006.

Keynote Speaker, “For the Greater Good: A Positive Philosophy of Markets,” Academy of Marketing Annual Conference/AMA Global Conference, Dublin, Ireland, July 2005.

“The New Femininity,” Leo Burnett, Chicago, June 2005.

“The New Femininity,” Procter & Gamble, Cincinnati, April 2005.

“Postindustrial Pictography,” Oxford University, April, 2005.

“Expressing the Self through Dress: Psychology and the Politics of Appearance,” Dept. of Psychology, University of Houston, April 18, 2005.

“Shocking Mom: American Girls in Historical Perspective,” Keynote Speaker, Women’s History Month, University of North Carolina, Wilmington, March 2005.

“Reappropriating Femininity: New Ideals and the Third Wave,” Bryn Mawr College, Sponsored by the Women’s Center and Gender Studies, February 2005.

“Visible Dreams: Inspiration, Aspiration, and Expression,” The New Femininity, Advertising Educational Foundation, New York, October 2004.

“Metatheory and Microtheory,” ACR Doctoral Consortium, Portland, Oregon, Fall 2004.

“History and Sociology in Consumer Behavior,” Research Series, Leeds School of Business, University of Colorado, March 2002.

Lecture Series on Marketing and Culture, University of Hannover, Germany, June 2000.

**Service**

Editor, Advertising and Society Review, published by Johns Hopkins University Press.

Conference Co-chair, Association for Consumer Research Gender Conference, Summer 2004.

Chair, How Does Advertising Shape the Image of Women? AEF, Chicago, October, 2003.

National Board of Directors, Advertising Educational Foundation, New York.

Co-chair, 19^th^ Annual Advertising and Consumer Psychology Conference, University of Michigan, Spring 2000. University of Michigan - Ann Arbor.

Co-editor for the advertising and marketing sections of the International Encyclopedia of the Social and Behavioral Sciences.

Conference Co-chair, Association for Consumer Research, for 1998 conference.

**Honors, Recognitions, Achievements, Grants**

Fell Fund Grant, July 2008, “Girls’ Education and Sanitary Care in Developing Countries.”

ESCR/DFID Poverty Reduction Grant, “Avon in Africa,” begins March 2008

Fell Fund Grants, March and June 2007, “Avon in Africa”

Fellow, Illinois Program for Research in the Humanities, 1999/2000.

Best Article Award, Journal of Advertising, 1997 and 1998.

Finalist, Best Article Award, Journal of Consumer Research, 1996.

Research Fellow, American Academy of Advertising, 1993.

Visiting Scholar, Smithsonian Institution, 1992.

University Fellow, The Graduate School of the University of Texas at Austin, 1989/90, 1990/1991.

Graduate, School of Criticism and Theory, Dartmouth College, 1990.

Jesse H. Jones Fellowship in Communication, University of Texas at Austin, 1988/89.

**Catherine S. Dolan**

Curriculum Vitae

**Employment**

2007- University Lecturer, Marketing, Culture and Society, Saïd Business School, University of Oxford, UK

2003-2007 Assistant Professor, Department of Sociology and Anthropology, Northeastern University, Boston, MA, USA

1999-2002 Lecturer & Co-Director of Masters in Gender Analysis of Development, School of Development Studies, University of East Anglia, Norwich, UK

1999 Visiting Lecturer, Department of Anthropology and Sociology, School of Oriental and African Studies, University of London, UK

1998-1999 Research Officer, Institute of Development Studies, University of Sussex, Brighton, UK

1992-1994 Assistant Project Manager, Institute for Development Anthropology, Binghamton, NY

**relevant research grants**

2008 John Fell Fund, Oxford University, ‘Girl’s Education and Sanitary Care in Developing Countries’, Joint Research Grant with A. Berlan, S. Dopson, P. Montgomery and L. Scott

2008 Templeton College, ‘Tampons for Africa: Feminine Hygiene and Educational Outcomes among Adolescent Girls’, Joint Research Grant with A. Berlan, S. Dopson, P. Montgomery and L. Scott

2007 ESRC/DFID, ‘Avon in Africa: Reducing Poverty through Global Exchange’, Joint Research Grant with Dr L. Scott

2007 John Fell Fund, Oxford University, ‘Avon in Africa: Women, Trading Circles, and Economic Development: Part II’, Joint Research Grant with Dr L. Scott

2007 John Fell Fund, Oxford University, ‘Avon in Africa: Women, Trading Circles, and Economic Development’, Joint Research Grant with Dr L. Scott

2006 National Science Foundation, Cultural Anthropology Program, **‘**Tracing the Socioeconomic Effects of Fairtrade in Kenya**’**

2002-2004 Social Science Research Grant, UK Department for International Development, Joint Research Grant with Dr S. Barrientos (IDS) and Dr A. Tallontire (NRI), ‘Ethical Trade: Gender, Rights and Participation’ (South Africa, Kenya, Zambia)

2000-2003 Economic and Social Research Grant, UK Department for International Development, Joint Research Grant between nine researchers at Institute of Development Studies and University of East Anglia, ‘Globalization, Production and Poverty’ (Kenya, South Africa, Bangladesh, Vietnam)

**education**

1998 PhD, Anthropology, State University of New York at Binghamton

1993 MA, Anthropology, State University of New York at Binghamton

1982 BA, Anthropology, University of Colorado at Boulder

1994 Certificate, Kiswahili, Summer Program in African Languages, Cornell University

**selected publications**

**Book**

2006 Barrientos, Stephanie and Catherine Dolan (eds.), Ethical Sourcing in the Global Food System*,* London: Earthscan.

**Journal Articles**

In press Dolan, Catherine, ‘Arbitrating Risk through Moral Values: The Case of Kenyan Fair Trade’, in Hidden Hands in the Market: Ethnographies of Fair Trade, Ethical Consumption, and Corporate Social Responsibility, Research in Economic Anthropology 2008, (28): 271–296.

2008 Dolan, Catherine, ‘The Mists of Development: Fairtrade in Kenya Tea Fields’, Globalizations 5(2): 1-14.

2008 Blowfield, Mick and Catherine Dolan, ‘Stewards of Virtue?: The Ethical Dilemma of CSR in African Agriculture’, Development and Change 39(1): 1-23.

2007 Dolan, Catherine, ‘Market Affections: Moral Encounters with Kenyan Fairtrade Flowers’, Ethnos 72(2): 239–261*.*

2005 Dolan, Catherine, ‘Fields of Obligation: Rooting Ethical Consumption in Kenyan Horticulture’, Journal of Consumer Culture 5(3): 365-389.

2005 Dolan, Catherine, ‘Benevolent Intent: The Development Encounter in Kenya’s Horticulture Industry’, Journal of Asian and African Studies 40(6): 411-437.

2005 Dolan, Catherine and Maggie Opondo**,** ‘Seeking Common Ground: Multistakeholder Initiatives in Kenya’s Cut Flower Industry’, Journal of Corporate Citizenship (18): 87-98.

2005 Tallontire, Anne, Dolan, Catherine, Barrientos, Stephanie and Sally Smith, ‘Reaching the Marginalised? Gender, Value Chains and Ethical Trade in African Horticulture’, Development in Practice 15(3&4): 559-571.

2004 Dolan, Catherine, ‘‘I Sell My Labor Now’: Gender and Livelihood Diversification in Uganda’, Canadian Journal of Development Studies 25(4): 665-683.

2004 Dolan, Catherine, ‘On Farm and Packhouse: Employment at the Bottom of a Global Commodity Chain’, Rural Sociology 69(1): 99-126.

2001 Dolan, Catherine, ‘The Good Wife: Struggles over Land and Labour in the Kenyan Horticultural Sector’, Journal of Development Studies 37(3): 39-70.

**recent presentations**

2008 ‘Virtual Moralities: The Mainstreaming of Fairtrade in Kenyan Tea Fields’, *Andrew W. Mellon Foundation Seminar Series*, The Fate of Food: Agricultural Production in the Fourth World Food Regime, University of North Carolina, Chapel Hill, April 25-26.

2008 ‘Corporate Evangelism: Corporate Evangelism: Avon Trading Circles and Gender Empowerment in South Africa’, *ESRC Research Seminar*: Informalisation and Risk: The Growth of Precarious and Contingent Work, April 4.

2008 ‘Whose Development? Transparency, Empowerment and Partnership in Kenyan Fair Trade Fields’, *Oxford Achilles Seminar*, February 11.

2007 ‘The Fog of Development: Fairtrade in Kenyan Tea Fields’, Panel on What's Fair?’, Environmental and Social Justice Through Markets, 20*07 American Anthropological Association Annual Meeting*, Washington, DC, November 28-December 1.

2006 ‘Fields of Obligation’, Invited Presentation, Colloquium Series: Cultures and Commodities, *Department of Anthropology, University of Kentucky*, Lexington, KY, September 22.

2006 ‘A Slave Has No Justice: Gender and Ethical Relevance in Kenya’s Cut Flower Industry’, Paper Prepared for XVI ISA *World Congress of Sociology*, Durban, South Africa, July 24.

2006 ‘Virtue at the Checkout Till: Salvation Economics in Kenyan Flower Fields’, Invited Paper for Panel: Spirits of Capitalism: Translations of Moral Value into Market Efficiency, *Society for Cultural Anthropology Annual Meeting*, Milwaukee, WI, May 5-6.

2005 ‘‘Pregnancy is in the Stomach, not in the Hands’: Gender, Health, and Well-Being in Kenya’s Cut Flower Industry Guilt’, Paper Presented at *African Studies Association Annual Meeting*, Washington, DC, November 17–20.

| Name | Dr | Sue | Dopson |
| --- | --- | --- | --- |
| Date of birth | 28^th^ March, 1961 | | |
| Degree subject/  Professional Qualification | 1994 PhD in Healthcare Management, Leicester University.1984 MSc in Medical Sociology, Bedford College, University of London.1982 BSc in Sociology, Leicester University. | | |
| Present & Previous Positions held | 2001 – Present Rhodes Trust University Reader in Organisational Behaviour, Said   Business School, University of Oxford.2001 – 2005 Vice President, Templeton College, University of Oxford.1998 – 2001 Senior Tutor, Templeton College, University of Oxford.1999 – Present Visiting Professor in Organisational Behaviour, Amos Tuck School   of Business, Dartmouth College & MIT.1997 – Present Director of the Oxford Health Care Management Institute,   Templeton College.1994 – Present University Lecturer & Fellow, Templeton College, Said Business   School, University of Oxford.1990 – 1994 Research Fellow, Templeton College.1985 – 1990 Research Associate, Templeton College, University of Oxford. | | |
| Relevant Publications | Dopson, S. (1997). *Managing Ambiguity and Change: The Case of the NHS*. Macmillan. (Winner of the Emantel international sociology prize).Dopson, S. Fitzgerald, L. Ferlie, E. (2002). No magic targets! Lessons from UK studies of attempts to change clinical practice to become more evidence based. *Health Care Manage Review*. 27, pp 35–47.Dopson, S. and Mark, A. (2003). *Leading Health Care Organisations*. Palgrave Press.Dopson, S. (2005). The Diffusion of Medical Innovations: Can Figurational Sociology Contribute? *Organization Studies.* 26, pp 1125-1144.Dopson, S. and Fitzgerald, L. (2005). *Knowledge to Action*. OUP.Runner up of theBritish Socilogy Book award | | |
| Current & Recent Research Grants | 2006 – 2008 Study of networks in health care funded by the SDO.2003 – 2007 A study of the genetics knowledge parks and the Oxford genetics   knowledge park looking at the barriers to translation of genetic   knowledge. Funded by the Deartment of Halth as part of the GKP   funding to Oxford. £5 million. 2007- 2009 The role of support workers in the NHS funded by the SDO | | |

##

**Paul Montgomery**

Curriculum Vitae

**Personal Details**

**Surname** Montgomery  **Forename** Paul

**Date of Birth** 10 May 1962 **Nationality** British

**Department Address** Centre for Evidence Based Intervention

Barnett House, 32 Wellington Square, Oxford OX1 2ER

Tel 44 (0)1865 270325

**Professional and Academic Qualifications:**

**D.Phil** Brief treatment for child sleep disorders University of Oxford 2001

**MSc**  Applied Social Studies University of Oxford 1998

**DipSW** Social Work University of Oxford 1998

**BA (Hons)** Economics and Geography with Education University of Keele 1984

**Posts Held**

*2008 - present* University Reader in Psycho-Social Intervention, Centre for Evidence Based Intervention, University of Oxford, Barnett House, 32 Wellington Square, Oxford OX1 2ER.

*2006 – present* Fellow of Templeton College Oxford.

*2006 - 2008* University Lecturer in Evidence Based Intervention, Centre for Evidence Based Intervention, University of Oxford, Barnett House, 32 Wellington Square, Oxford OX1 2ER.

*2003 - 2006* Departmental Lecturer in Evidence Based Intervention, Department of Social Policy and Social Work, University of Oxford, Barnett House, 32 Wellington Square, Oxford OX1 2ER.

*2002 - 2003* (0.5 WTE) Clinic Social Worker, Aylesbury Child and Family Psychiatric Service, Sue Nicholls Centre, Manor House, Bierton Road, Aylesbury, HP20 1EG.

*1998 - 2004* (1.0 until 2002 then 0.5 WTE) Research Officer, University of Oxford Department of Psychiatry (Child and Adolescent Section), Park Hospital for Children, Old Road, Headington, Oxford, OX3 7LQ.

**Grants**

*The DOLAB Trial (DHA Oxford Learning and Behaviour). A randomised controlled trial.*

Martek Biosciences. £647,000

*Girls Education and Sanitary Care in Underdeveloped Countries. A pilot study*

Co-PI, (Lead PI is Prof Linda Scott Said Business School)John Fell Fund, University of Oxford £37,000

*Unaccompanied Asylum Seeking Children: A Pilot Study – Methodology and intervention*

John Fell Fund, University of Oxford £88,000

*A Series of Systematic Reviews of Personal Carers*

Swedish Board of Health and Welfare  £150,000

*A Series of Systematic Reviews of Social Care*

Danish Social Research Institute £90,000

*Randomised Trial of a Media-Based Parenting Intervention*

Lead – PI, Co - PI is Dr Frances Gardner, Oxford University Dept of Social Policy and Social Work)

Oxford University Research Development Fund and Oxfordshire Mental Healthcare Trust Charitable Fund

*Interventions for physical abuse in children: A series of systematic reviews*

Lead – PI, Co PIs are: Dr Frances Gardner (Oxford University Dept of Social Policy and Social Work) and Dr Paul Ramchandani (Oxford University Dept of Psychiatry).

Department for Education and Skills £106,000

*Melatonin for sleep disturbance in children with learning disabilities: A multi-centre randomised placebo-controlled trial*

PI of the Oxford site in collaboration with: Dr Richard Appleton (Alder Hey Children’s Hospital Liverpool); Dr Paul Gringas (St Thomas’ Hospital London); Dr Luci Wiggs (Oxford Brookes University)

Department of Health R&D Health Technology Assessment Programme £441,061

*A Systematic Review of Fish Oil for Bipolar Disorder*

ViFAB- Danish Complementary Medicine Board £8,000

**Most Recent Publications**

**Peer reviewed journal articles**

Spoormaker, V. I. and P. Montgomery (2008). "Disturbed sleep in post-traumatic stress disorder: Secondary symptom or core feature?" *Sleep Med Rev 12(3): 169-84.*

Austen, A. Bronstein, R. and Montgomery, P. Helping Asylum Seeking Children. *Community Care. April 16 2008 http://www.communitycare.co.uk/Articles/2008/04/16/107924/helping-unaccompanied-asylum-seeking-children.html*

Austen, A. Bronstein, R. and Montgomery, P. *A novel multi-component intervention for Unaccompanied Asylum-Seeking Young People: The Well Being Project- London Borough of Hillingdon. http://www.communitycare.co.uk/Articles/2008/05/09/107933/a-novel-multi-component-intervention-for-unaccompanied-asylum-seeking-young-people-the-well-being.html*

Burton, J., Montgomery, P., Mayo-Wilson, E., Underhill, K., and Thorgaard, C. (2007), Home visits for prevention of cognitive and functional impairment and for prolonging survival in non-demented elderly people. (Protocol). The Campbell Collaboration Library

Cyhlarova, E. Montgomery, P., Ross, M.A. Richardson, A.J. (2007) Niacin skin test response in dyslexia. Prostaglandins, Leukotrienes and Essential Fatty Acids 77, 123–128

Lilly, J. and Montgomery, P., (2007). Effects of Preparatory Courses on University Entrance Exams in High School-Age Students (Protocol). The Campbell Collaboration Library, http://www.campbellcollaboration.org/frontend2.asp?ID=129

Underhill, K., Operario, D., and Montgomery, P., (2007) Systematic review of abstinence-plus HIV prevention programs in high-income countries. PLoS Medicine 4(9): e275, doi:210.1371/journal.pmed.0040275.

Underhill, K., Operario, D., and Montgomery, P., (2007) Sexual abstinence only programmes to prevent HIV infection in high income countries: Systematic review. British Medical Journal; 335(7613):248.

Underhill, K., Operario, D., and Montgomery, P., (2007) Reporting deficiencies in trials of abstinence-only programmes for HIV prevention. AIDS; 21(2):266-268.

Stores, G., Montgomery, P., and Wiggs, L., (2006) The Psychosocial Problems of Children with Narcolepsy and Those With Excessive Daytime Sleepiness of Uncertain Origin Pediatrics 118; 1116-1123

Montgomery, P., Donkoh, C., and Underhill, K., (2006) Independent living programs for young people leaving the care system: The state of the evidence, Children and Youth Services Review 28 1435–1448.

Montgomery, P., Bjornstad, G., and Dennis, J., (2006) Media-based behavioural treatments for behavioural problems in children. The Cochrane Database of Systematic Reviews, Issue 1. Art. No.: CD002206. DOI: 10.1002/14651858.CD002206.pub3.

Hockney, R.A., Montgomery, P., Williams, C., Geddes, J., Cowen, P., (2006) The Lack of Effect of Chromium Supplementation on Mental State and Body Weight in People With Schizophrenia. Journal of Clinical Psychopharmacology. 26 (5).

**GHANA HEALTH SERVICE**

**ETHICAL REVIEW COMMITTEE ON RESEARCH INVOLVING HUMAN SUBJECTS (ERCRIHS)**

**(CHECKLIST**)

PI NAME: **Linda Scott**

Project ID (To be given by the Secretariat

| **Title of Project:** *Girls’ Education and Sanitary Care in Developing Countries: An Exploratory Study* | | **PI TO COMPLETE** |  | |  |  |
| --- | --- | --- | --- | --- | --- | --- |
|  |  | **No**  **N/A** |  | |  |  |
|  | | **Vulnerable/High Risk Group** |  | |  |  |
| 1 | | Is a vulnerable population being studied? | | |  | |
|  | | If yes, tick the vulnerable population being studied? |  | |  |  |
|  | | Pregnant women | | | Prisoners  Persons with mental or  Behavioural disorders  Others |  |
| 2 | | Is the justification for studying this vulnerable population adequate? |  | | |  |
| 3. | | Have adequate provisions been made to ensure that the vulnerable population is not being exploited? | | |  |  |
| **Responsible Technical Officer’s Comments:** | |  |  | |  |  |
| **Scientific and Technical Issues** | |  |  | |  |  |
| 1. | | Is the rational for the study clearly stated in the context of present knowledge? | | |  |  |
| 2. | | Is the hypothesis to be tested fully explained? | | |  |  |
| 3. | | Is the project design scientifically sound? | | |  |  |
| 4. | | Where present, is the control arm adequate? | | |  | |
| 5. | | Are the inclusion and exclusion criteria complete and appropriate? |  | |  |  |
| 6. | | Are the types and methods for subject allocation appropriate? | | |  |  |
| 7. | | Are the procedures for participant recruitment, admission, follow up and completion appropriate? |  | |  |  |
| 8. | | Are the drugs and/or devices to be used fully described? | | |  | |
| 9. | | Does the project design include appropriate criteria for stopping and discontinuing the research? | | |  |  |
| 10. | | Are the clinical procedures to be carried out fully described and appropriate? |  | | |  |
| 11. | | Are the laboratory tests and other diagnostic procedures fully described and appropriate? | | |  | |
| 12  13. | Is the Statistical basis for the study design appropriate and is the plan for analysis of the data appropriate?  Has the Protocol undergone scientific review?  (if applicable please provide evidence) | | |  | | |
| R**esponsible Technical Officer’s Comments:** | |  |  | |  |  |

**GHANA HEALTH SERVICE**

**ETHICAL REVIEW COMMITTEE ON RESEARCH INVOLVING HUMAN SUBJECTS (ERCRIHS)**

**CHECKLIST**

**PI NAME: Linda Scott**

Project ID (To be given by the Secretariat)

|  | | **PI TO COMPLETE** | |  | |  |  |
| --- | --- | --- | --- | --- | --- | --- | --- |
|  |  | **No** | | **N/A** | |  |  |
| **Informed Consent, Decision-making & Confidentiality** | |  | |  | |  |  |
| **1.**  Is the information sheet free of technical terms, written in laypersons’ language, easily understandable, complete & adequate? | |  | |  | |  |  |
| **2.**  Does it make it clear that the proposed study is research? | |  | |  | |  |  |
| **3.**  Does it explain why the study is being done and why the subject is being asked to participate | |  | |  | |  |  |
| 4. | | Does it clearly state the duration of the research**?** | | | |  |  |
| **5.**  Does it provide participants with a full description of the nature, sequence and frequency of the procedures to be carried out? | |  | |  | |  |  |
| **6.**  Does it explain the nature and likelihood of anticipated discomfort or adverse effects, including psychological and social risks, if any-and what has been done to minimize these risks, and the action to be taken if they occur? | |  | |  | |  |  |
| 7. | | Does it outline the possible benefits, if any, to the research participants | | | |  |  |
| 8. | Does it outline the possible benefits, if any, to the community or to society? | |  | | | | |
| 9 | | If confidentiality is not possible due to the research design, has this been conveyed to all relevant persons? | | | |  |  |
| 10 | | Does it inform the research participants that their participation is voluntary and refusal to participate (or discontinue participation) will involve no penalty or loss of medical benefits to which the participant was otherwise entitled? | | | |  |  |
| 11. | | Does it describe the nature of any compensation or reimbursement to be provided? | | | |  |  |
| 12 | | Does it provide the alternatives to participation? | | | |  | |
| 13. | | Does it provide the name and contact information of a person who can provide more information about the research project at any time? | | | |  |  |
| 14. | | Has provision been made for subjects incapable of reading and signing the written consent form (e.g. illiterate patients)? (Please attach) | | | |  |  |
| 15 | Does it conclude with a statement such as ‘’I have read the foregoing information, or it has been read to me. I have had the opportunity to ask questions about it and any question I have asked have been answered to my satisfaction. I consent voluntarily to participate as a subject in this study and understand that I have the right to withdraw from the study at any time without in any way it affecting my further medical care’’ | | | |  | | |

**GHANA HEALTH SERVICE**

**ETHICAL REVIEW COMMITTEE ON RESEARCH INVOLVING HUMAN SUBJECTS (ERCRIHS)**

**CHECKLIST**

**PI NAME: Linda Scott**

**Project ID (To be given by the Secretariat**

|  | PI TO COMPLETE |  |  |  |
| --- | --- | --- | --- | --- |
|  | No | N/A |  |  |
| 16. | Does it provide information to the research participants on the costs to the participants involved in terms or time, travel, man-days lost from work, etc. and reimbursements, if any? | |  |  |
| 17. | Has provision been made for subjects incapable of giving personal consent (e.g. for cultural reasons, children or adolescents less than the legal age for consent in the country in which research is taking place, subjects with mental illness, etc)? (Please attach). | |  |  |
| 18. | Does it outline the procedure that will be followed to keep participants informed of the progress and outcome of the research? | |  |  |
| **Responsible Technical Officer’s Comments:** |  |  |  |  |
| **Other materials, documents and study instruments (Patient recruitment material, Questionnaires** |  |  |  |  |
| 1 | Is the Participant Recruitment Material (e.g. advertisements, notices, media articles, transcripts of radio messages) provided both in English and in the local language? | |  |  |
| 2. | Do these materials make claims that may not be true? | |  |  |
| 3. | Do they make promises that may be inappropriate in the research setting (e.g. provide undue incentives or emphasize remuneration? | |  |  |
| 4. | Does the study involve questionnaires, diaries, study instrument? | |  |  |
| 5. | Are these attached to the proposal (In **English** and local language)? Will be transcribed | |  |  |
| 6. | Are the questionnaires written in lay language and easily understood? | |  |  |
| 7. | Are the questionnaires relevant to answer the research question? | |  |  |
| 8. | Are the questionnaires worded sensitively? | |  |  |
| 9. | Does the consent information and form describe the nature and purpose of the questions to be asked? | |  |  |
| 10. | If applicable, does the consent information and form make it clear that some of the questions may prove embarrassing for the participant? | |  |  |
| 11. | Does the proposal describe how confidentiality of the questionnaires will be maintained (i.e. will they be coded or anonimized)? | |  |  |
| 12. | Does the consent information and form state that the participant is free to not answer any question? | |  |  |
| 13. | Where applicable, does the informed consent form make it clear that the in-depth interview or focus group discussion is likely to be audio or video taped? | |  |  |
| 14. | Where applicable, does the consent form mention how and for how long these tapes are going to be stored? | |  |  |
| **Responsible Technical Officer’s Comments:** |  |  |  |  |

**GHANA HEALTH SERVICE**

**ETHICAL REVIEW COMMITTEE ON RESEARCH INVOLVING HUMAN SUBJECTS (ERCRIHS)**

**(CHECKLIST)**

**PI NAME: Linda Scott**

**Project ID (To be given by the Secretariat**)

|  |  |  |  |  |
| --- | --- | --- | --- | --- |
|  | No | N/A |  |  |
| Clinical Trials |  |  |  |  |
| 1. | Is this a new drug or vaccine trial? | |  |  |
| 2. | If applicable, is clearance from the national drug regulatory authority attached? * | |  |  |
| 3. | Is the Investigator’s Brochure (including safety information) attached? | |  |  |
| 4. | Is the Adverse Drug Reaction/Adverse Event Reporting form attached? | |  |  |
| 5. | Has a Data Safety Monitoring Board been established | |  |  |
| 6. | Are the names of the chairperson and members of the DSMB available for the records? | |  |  |
| Responsible Technical Officer’s Comments: |  |  |  |  |
| **Human Biological Materials** |  |  |  |  |
| 1, | Will human biological materials (tissues, cells, fluids, blood, genetic material or genetic information) be collected as part of the research? | |  |  |
| 2. | Does the consent information and form fully describe the nature, number and volume of the samples to be obtained and the procedures to be used for obtaining them? | |  |  |
| 3. | Does the consent information and form indicate if the procedures for obtaining these materials are routine or experimental and if routine, are more invasive than usual? | |  |  |
| 4. | Does the consent information and from clearly describe the use to which these samples will be put? | |  | |
| 5. | Does the consent information and form include the provision for the subject to decide on the use of left-over specimens in future research of a restricted, specified or unspecified nature? | |  |  |
| 6. | Does the consent information and form cover for how long such specimens can be kept and how they will be finally destroyed? | |  |  |
| 7.  Does the proposal describe how specimens will be coded/anonimized |  | |  |  |
| 8. | Where applicable, does the consent form mention that genetic testing/genomic analysis will be carried out on the human biologic materials? | |  |  |
| **Responsible Technical Officer’s Comments** |  |  |  |  |

**Budget Outline**

| **Item** | **Cost (£)** |
| --- | --- |
| Air fare and subsistence (hotel, meals) | 18,550 |
| Vaccination, visas | 1,100 |
| Equipment/data services | 805 |
| Research assistance | 5,200 |
| Field costs | 9,450 |
| **Total (£)** | 35,105 |
| **Total (GHc)** | 72,302 |

**Project Sponsor**

This project has been funded by the John Fell Oxford University Press Research Fund. The grant period is from September through November, 2008
